# *Staphylococcus aureus* isolates colonizing and infecting cirrhotic and liver-transplantation patients: comparison of molecular typing and virulence factors

**DOI:** 10.1186/s12866-015-0598-y

**Published:** 2015-11-14

**Authors:** Larissa Marques de Oliveira, Inneke Marie van der Heijden, George R. Golding, Edson Abdala, Maristela P. Freire, Flavia Rossi, Luiz C. D’ alburquerque, Anna S. Levin, Silvia F. Costa

**Affiliations:** Department of Infection Control, Hospital das Clínicas, University of São Paulo, São Paulo, Brazil; Laboratory of Medical Investigation 54 (LIM-54), Hospital Das Clínicas FMUSP, São Paulo, Brazil; National Microbiology Laboratory, Winnipeg, Manitoba Canada; Liver Transplantation Unit, Hospital das Clinicas, University of São Paulo, São Paulo, Brazil; Laboratory of Microbiology, Hospital das Clinicas, University of São Paulo, São Paulo, Brazil; Department of Infectious Diseases, LIM-54, Faculdade de Medicina, University of São Paulo, Avenida Doutor Enéas de Carvalho Aguiar, 470, São Paulo, SP 05403-000 Brazil

**Keywords:** Methicillin-resistant *Staphylococcus aureus*, Colonization, *spa* typing, PFGE, MLST

## Abstract

**Background:**

*S. aureus* is an important agent of colonization and infection in liver transplant patients. It harbors several virulence factors that can increase its pathogenicity. However, studies of virulence and molecular typing of MRSA in cirrhotic and liver transplantation patients are scarce.

**Results:**

Here we use SCC*mec*, PFGE, *spa* typing, MLST and virulence factors to characterize MRSA isolates in pre and post liver transplantation patients. Sixteen (13 %) of 126 cirrhotic and 15 of the 64 liver-transplanted patients (23 %) were colonized by MRSA (*p* = 0.091). SCC*mec* types I, II and III that are generally associated with nosocomial infections were identified in 91 % of the isolates. None of the isolates carried PVL, adhesion factors and *fi*b gene. Only three MRSA colonized isolates carried *tst* gene and were characterized as SCC*mec* type I and t149. Ten *spa* types and five STs were identified; t002 and ST105 were the most frequent profiles. *Spa* types and ST1510 never described in Brazil and a new *spa* type t14789 were identified. Nineteen PFGE subtypes were found and grouped into nine types. There was a predominant cluster, which was related to the New York/Japanese epidemic clone and harboured SCC*mec* type II identified in both cirrhotic and post-transplantation patients. Based on SCC*mec* and virulence factors the MRSA isolates belonged to NY/Jpn clone seen be more similar to the USA100 MRSA isolates.

**Conclusions:**

Although without significance, liver-transplantation was more frequently colonized by MRSA than cirrhotic patients. The most frequent SCC*mec* was type II, and the predominant cluster was related to the New York/Japanese clone. A new *spa* t14789, and ST1510 never reported in Brazil were identified.

## Background

Methicillin-resistant *Staphylococcus aureus (*MRSA) infections rates have been increasing rapidly worldwide over the past few decades [1]. MRSA is currently endemic in many hospitals in several countries [1]. According to a SENTRY study, in Brazil*,* the rates of MRSA infections are ~30 % and *S. aureus* is responsible for ∼ 20 % of nosocomial primary bloodstream infections (BSIs) [2]. MRSA is an important cause of surgical infection in liver transplantation patients, increased duration of hospitalization, costs, morbidity and mortality [3]. Therefore, it is of great importance to understand the epidemiology of MRSA in this population of patients.

Cirrhotic patients are more frequently colonized by MRSA than the general population and the burden of infectious diseases pre and post liver transplantation is clearly attributable to the dysfunction of defensive mechanisms of the host, because of cirrhosis, as well as because of the use of immunosuppressive agents [4]. Several virulence factors present in *S. aureus* that allow the adherence, colonization and ability to invade tissues can increase the pathogenicity of this microorganism, among them the Panton-Valentine leucocidin (PVL), a toxin that can be able to make pores in the polymorphonuclear cells [5]. However, studies of virulence of MRSA in cirrhotic and liver transplantation patients are scarce.

Molecular methods such as the characterization of Staphylococcal Chromosome Cassette *mec* (SCC*mec*) by polymerase chain reaction (PCR), Multi Locus Sequence Typing (MLST), Pulsed-Field Gel Electrophoresis (PFGE) and *Staphylococcus* protein A (*Spa*) typing had been developed to evaluate MRSA isolates and to develop strategies to control and prevent colonization and infection. SCC*mec* typing is a rapid and easy technique essential for understanding the molecular epidemiology of MRSA [6].

Another widely used tool is PFGE that has an excellent discriminatory power and is very useful for epidemiological studies although it is a laborious and time consuming technique [7]. *Spa* typing is a more recent method based on sequencing the polymorphic region X or short-sequence repeat (SSR) region of the gene *spa*. Changes in the SSR region arise due to deletions, duplications and point mutations, which results in a diverse collection of ‘*spa* types’, where each spa type consists of a specific combination of SSRs [8]. *Spa* typing has been shown to be useful not only for investigation of hospital outbreaks but also for studies of molecular evolution of MRSA [8]. The major advantages of *spa* typing include it is fast, easy to use, has unambiguous data interpretation and has a standard nomenclature database.

Some studies observed a high concordance between *spa* types and PFGE epidemic clones [9, 10]. Golding and colleagues showed that the same *spa* types correspond to multiple PFGE epidemic clones and require additional molecular typing to discriminate them. These authors proposed the use of Panton-Valentine leukocidin and SCC*mec* typing to differentiate between MRSA isolates that shared the same *spa* type but presented multiple PFGE epidemic types [10]. However, PVL characterization was not able to differentiate between isolates associated with multiple PFGE epidemic types, although it can be used as a general indicator of PFGE epidemic type.

The aim of this study was to compare the use of molecular methods (SCC*mec*, PFGE, *spa* typing and virulence factors) to characterize MRSA isolates that colonized and causing infection in patients before (cirrhotic) and after liver transplantation.

## Results

### Study population

One-hundred and twenty-six outpatients with liver diseases on list waiting for liver transplantation and 64 liver-transplanted patients were evaluated. According to chart reviews and the questionnaire applied to the 126 listed patients, all patients of list transplantation reported go to the hospital at least once a month for doctor visits or routine tests since the discovery of the disease. Furthermore, 47 (37 %) reported have been hospitalized in the previous 6 months. In addition, 30 (24 %) patients reported previous use of antimicrobials, 58 (46 %) reported not having used any antimicrobial previously and 38 (30 %) did not remember about the use of these drugs in the last 6 months. All liver-transplanted patients had a 48-hour course of ampicillin plus cefotaxime as antimicrobial prophylaxis and received tacrolimus and steroid as imunossuprevise therapy. Those that developed kidney failure had the therapy replaced by adjusted doses of tacrolimus plus mycophenolate mofetil (MMF) and steroid.

Demographic and clinical data of the study population is shown in Table 1. Sixteen patients of the 126 listed outpatients (13 %) and 15 of the 64 liver-transplanted patients (23 %) were colonized by MRSA (*p* = 0.091). Fourteen patients of list of waiting for liver transplantation were transplanted during the study and were included between the 64 liver-transplanted patients. Among then, two were colonization in pre and post transplantation period and three were colonization only in post transplantation period.

**Table 1:** Clinical and demographic characteristics of 190 patients before and after liver transplantation undergoing screening for methicillin-resistant *S. aureus* (MRSA)

Characteristics	Cirrhotic Patients on the waiting list for liver transplantation (126)	Liver-transplanted patients (64)
Male	85 (67 %)	37 (58 %)
Age (years)		
Median	53	52
Range	19–71	17–71
Underlying diseases^a^		
Cirrhosis due to hepatitis C	38 (30 %)	10 (16 %)
Alcoholic cirrhosis	23 (18 %)	8 (12.5 %)
Cryptogenic cirrhosis	13 (10 %)	9 (14 %)
Auto immune hepatites	9 (7 %)	2 (3 %)
Cirrhosis due to hepatitis B	8 (6 %)	3 (5 %)
Sclerosing cirrhosis	5 (4 %)	1 (1.5 %)
Primary biliary cirrhosis	4 (3 %)	1 (1.5 %)
Secondary biliary cirrhosis	3 (2 %)	2 (3 %)
Nash (nonalcoholoic steatohepatitis)	3 (2 %)	1 (1.5 %)
Fulminant hepatites	-	4 (6 %)
hepatocellular carcinoma	-	3 (5 %)
Unknown cause	4 (3 %)	14 (22 %)
Other^b^	14 (11 %)	8 (13 %)
Site of colonization by MRSA		
Nasal only	8 (50 %)	9 (60 %)
Groin only	2 (12.5 %)	2 (13 %)
Nasal and groin	6 (37.5 %)	4 (27 %)

^a^The total is greater than 100 % because some patients had more than one disease

^b^Other diseases related: Caroli’s syndrome, schistosomiasis, *alpha*-*1 antitrypsin deficiency, Wilson’s syndrome,* biliary atresia, Liver polycystic disease and familial amyloid polyneuropathy

Among 64 liver transplanted patients, five had infections by MRSA (two pneumonias, one infection related to central venous line, one blood stream infection and one peritonitis), three of them evoluted to death. Between them, three patients were colonized in post transplantation period and two patients were colonized in pre and post transplantation period. Only one patient with pneumonia had the same cluster NY/JP in colonization and infection. Twenty-eight (47 %) liver transplanted patients received steroid plus tacrolimus and 32 (53 %) MMF plus steroid and tacrolimus as immunosuppressive therapy, MRSA colonization was respectively 7 of 28 (46 %) and 8 of 32 (53 %) patients (*p* = 0.60), data of four patients were not available.

### Isolates

Forty-three isolates from 31 patients were identified as MRSA. All isolates were positive for the *mec*A and *co*A genes. All isolates were epidemiologically defined as HA-MRSA.

Only one isolate per patient was selected for further molecular typing. When patients had MRSA isolates from the both sites with the same PFGE, the nasal MRSA isolate was choose for next steps. Furthermore, one patient showed two phenotypically different MRSA isolates from the same site and both isolates were included on the study. Two patients were positives only in groin and these isolates were evaluated as well.

A total of 32 isolates were selected for further molecular typing, which included 16 isolates from patients of the liver-transplant waiting list and 16 isolates from liver-transplanted patients.

### SCC*mec* and virulence factors

Among the MRSA isolates, 6 (19 %) carried SCC*mec* I, 18 (56 %) SCC*mec* II, 5 (16 %) SCC*mec* III and 3 (9 %) SCC*mec* IVa. SCC*mec* types that are generally associated with nosocomial infections (SCC*mec* types I, II and III) were identified in 91 % of the HA-MRSA isolates. Of these, SCC*mec* type II was the most prevalent type. None of the isolates carried PVL, adhesion factors such as exfoliating A, B (*eta* and *etb* genes) and fibrinogen binding protein (*fi*b gene). Three MRSA isolates carried the *tst* gene, all isolates were characterized as SCC*mec* type I, belonged to cluster E and t0149 and were isolated from one cirrhotic patient and two liver-transplanted patients. None of patients carrying *tst* isolates developed infections.

### *Spa* typing

*Spa* typing of the 32 tested MRSA isolates revealed nine known *spa* types (t002, t010, t037, t088, t110, t149, t311, t539 and t3824) and one isolate from a cirrhotic patient was characterized as a new *spa* type t14789. This new *spa* type presented the following SSR: (r26, r23, r17, r16, r20, r17, r12, r17, r16), its present a deletion of repeat and a replacement of r34 by r16 comparing t002, and was closely related to t311 with a replacement of r34 by r16. Of these 9 *spa* types, t002 was the most common, representing 40.6 % of all isolates.

### PFGE

**Fig. 1:**
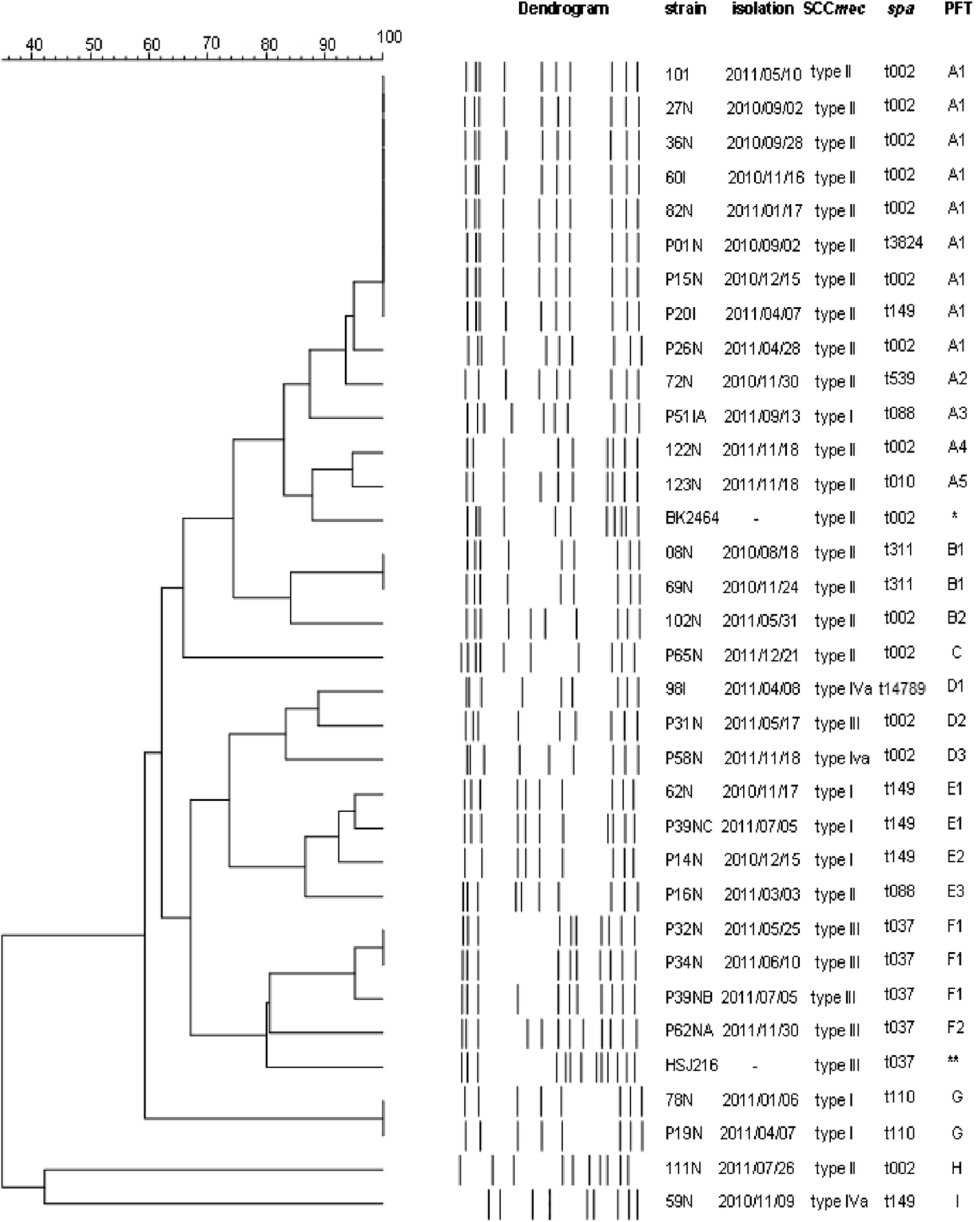
Molecular characteristics of 32 MRSA isolates of cirrhotic and liver transplanted patients from August 2010 to December 2011 (*The strain BK2464 is representative New York/ Japan epidemic clone. **The strainHSJ216 is representative Brazilian epidemic clone)

**Fig. 2:**
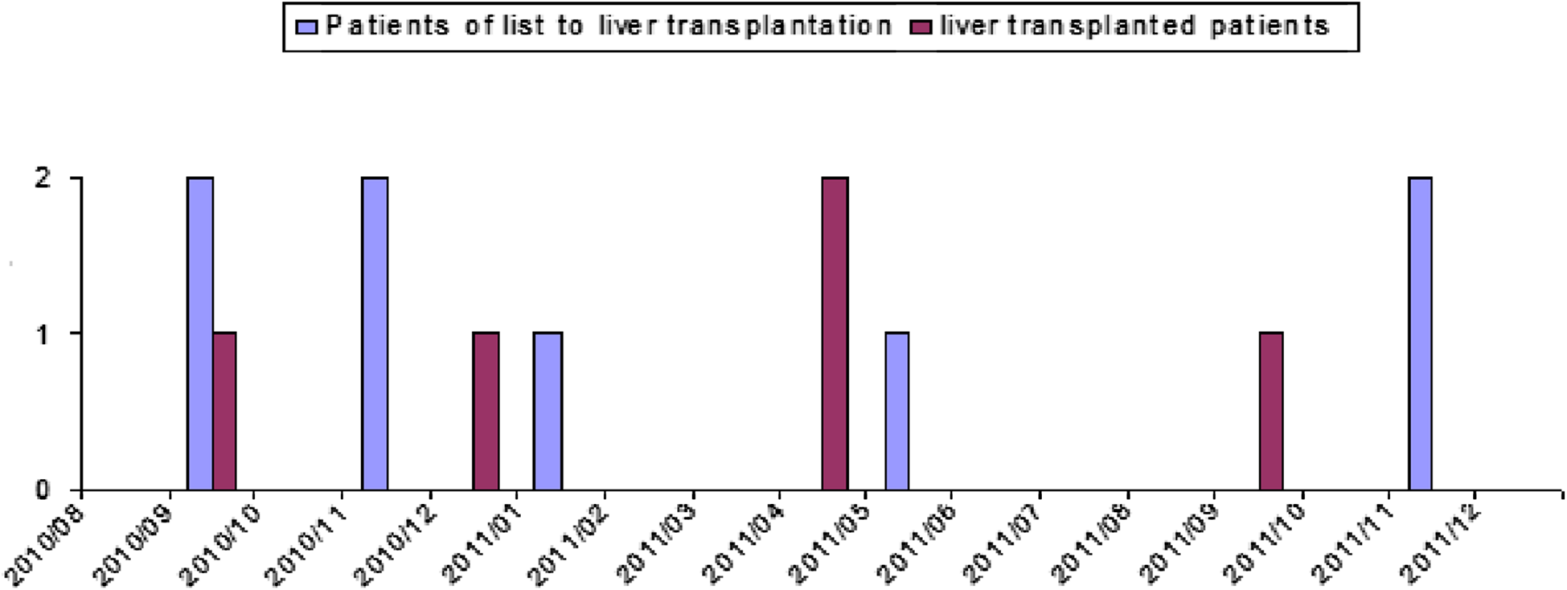
Distribution of MRSA strains belonged to the predominant NY/Japanese clone isolated from cirrhotic and post-transplantation patients over the study period, from 2010 to 2012

To define the Pulsed-Field types (PFT) obtained by PFGE, two cutoff levels were considered: >80 % to define types and 80–95 % to define subtypes. The types were represented by capital letters and subtypes by numbers. Between the 32 MRSA isolates, 9 PFGE types and 19 PFGE subtypes were identified. The dendrogram revealed that 13 (41 %) MRSA isolates belonged to a major cluster, the PFT A, which was related with the representative New York/Japan epidemic clone (NY/Jpn clone) BK2464, this predominant cloned was identified in cirrhotic outpatients (*n* = 8) and in post-transplantation patients (*N* = 5) and distributed over a year period (Fig. 2). Other smaller clusters were noted (PFT B, C D, E and F, G, H and I). The representative Brazilian epidemic clone (BEC) HSJ216 was related with 4 (12 %) MRSA isolates with PFT F, all them carried the same *spa* type and SCC*mec* type (t037-SCC*mec* III) and were from post transplantation patients. The dendrogram, SCC*mec*, *spa* type and PFT were represented in Fig. 1.

The distribution of SCC*mec*, *spa* type and PFGE profiles among the pre and post transplantation patients can be seen in Table 2. Distribution of MRSA strains belonged to the predominant NY/Japanese clone isolated from cirrhotic and post –transplantation patients over the study period can be seen in Fig. 2.

**Table 2:** Association between molecular typing profiles and epidemic clones, *spa* types, SCC*mec* types and virulence factors in methicillin-resistant *S. aureus* isolates of cirrhotic and liver transplanted patients from August 2010 to December 2011

PFT	Relationship with Epidemic Clones	Spa type (number of isolates)	SCC*mec* type	Genes for Virulence factors					
				PVL	TST	*luk*DE	*fn*PA	Cirrhotic	Liver transplantation
A1	NewYork/Japan clone	t002 (7)	II	(−)	(−)	(+)	(+)	5	2
A1	NewYork/Japan clone	t3824 (1)	II	(−)	(−)	(+)	(+)	-	1
A1	NewYork/Japan clone	t149 (1)	II	(−)	(−)	(+)	(+)	-	1
A2	NewYork/Japan clone	t539 (1)	II	(−)	(−)	(+)	(+)	1	-
A3	NewYork/Japan clone	t088 (1)	I	(−)	(−)	(+)	(+)	-	1
A4	NewYork/Japan clone	t002 (1)	II	(−)	(−)	(+)	(+)	1	-
A5	NewYork/Japan clone	t010 (1)	II	(−)	(−)	(+)	(+)	1	-
B1		t311 (2)	II	(−)	(−)	(+)	(+)	2	-
B2		t002 (1)	II	(−)	(−)	(+)	(+)	1	-
C		t002 (1)	II	(−)	(−)	(+)	(+)	-	1
D1		t14789 (1)	IVa	(−)	(−)	(+)	(+)	1	
D2		t002 (1)	III	(−)	(−)	(+)	(+)	-	1
D3		t002 (1)	IVa	(−)	(−)	(+)	(+)	-	1
E1		t149 (1)	I	(−)	(+)	(+)	(+)	1	-
E1		t149 (1)	I	(−)	(+)	(−)	(+)	-	1
E2		t149 (1)	I	(−)	(+)	(+)	(+)	-	1
E3		t088 (1)	II	(−)	(−)	(+)	(+)	-	1
F1	Brazilian clone	t037 (2)	III	(−)	(−)	(+)	(+)	-	2
F1	Brazilian clone	t037 (1)	III	(−)	(−)	(−)	(−)	-	1
F2	Brazilian clone	t037 (1)	III	(−)	(−)	(+)	(+)	-	1
G		t110 (2)	I	(−)	(−)	(+)	(+)	1	1
H		t002 (1)	II	(−)	(−)	(+)	(−)	1	-
I		t149 (1)	IVa	(−)	(−)	(−)	(−)	1	-

### MLST

Five Sequence types (ST) were identified. The results of the MLST for isolates of each PFGE type (A to I) are presented in Table 3.

**Table 3:** Methicilin-resistant *Staphylococcus aureus* isolates evaluated as to their MLST. Comparison of Multi Locus Sequence Typing with PFT and *Spa* typing of Methicilin-resistant *Staphylococcus aureus* isolates over study period, from August 2010 to December 2011

Isolates	Body site	*spa* type	SCC*mec*	ST^a^	PFT^b^
27 N	nasal	t002	II	ST105	A1
08 N	nasal	t311	II	ST105	B1
P65N	nasal	t002	II	ST105	C
98I	groin	t14789	IVa	ST1176	D1
62 N	nasal	t149	I	ST5	E1
P32N	nasal	t37	III	ST239	F1
78 N	nasal	t110	I	ST105	G
111 N	nasal	t002	II	ST105	H
59 N	nasal	t149	IVa	ST1510	I

^a^ *ST* sequence type, ^b^Pulsed-field typing

## Discussion

Previous studies have shown that MRSA colonization in cirrhotic patients varies from 16 to 28 % and that MRSA colonization can be associated with a high risk for infection in post liver transplantation patients [11–14]. In spite of this, few studies evaluated the molecular characterization of MRSA in liver transplantation patients, most of them described outbreaks, only *pvl* was evaluated among the virulence factors, and no study compared PFGE with *spa* typing [14–16]. To our knowledge, this is the first prospective study of molecular characterization of MRSA isolates in patients before and after liver transplantation.

MRSA decolonization has been recommending pre surgery in high risk patients such as cardiac and orthopedic patients. However, data in liver transplantation is scarce; most of studies of MRSA carriage in post liver transplanted patients were retrospective, evaluated only nares [13, 17, 18] and the efficacy of pre surgery decolonization need to be better addressed. In our study, a high proportion of swabbed patients were colonized with MRSA, and nares were the most frequent site of colonization. We did not identify outbreak due to MRSA in our hospital during the study period which might impact the colonization rates, however, MRSA screening is not performed as routine in the high risk patients such as intensive care patients, thus, MRSA colonization was underreported.

A previous study also showed that 236 nares (46 %) were more positives than rectal cultures (18 %). Based on our results it seems that there is no need to collected cultures of other sites besides nares to screening MRSA in cirrhotic or liver transplanted patient. Although not significant, in our study liver transplanted patients were more likely to be colonized by MRSA than cirrhotic patients (*p* = 0.091). Thirty-three percent of the MRSA isolates were from transplanted patients and 17 % of isolates were from cirrhotic patients. However, only three patients colonized developed infection due to MRSA post-transplantation, and one patient presented the same cluster NY/Jpn in colonization and infection. In contrast, during the period of this study, there were outbreaks by Gram-negative bacteria in the liver transplantation unit of our hospital [19]. Interesting, none of patients colonized with MRSA harboring *tst* gene developed infections. It seems that the high rate of MRSA colonization did not increase the rate of infection.

Regarding the molecular typing data we identified nine different Pulsed Field Types (A-I). The cluster A was predominant (41 %) and related to the NY/Jpn clone and present in cirrhotic and liver transplanted patients. Only 12.5 % of MRSA isolates belonged to BEC that was identified only in post transplanted patients. Nine known *spa* types and a new *spa* typing (t14789) were identified. Among them, *spa* type t002 was the most frequent, six *spa* types were associated with the New York/Japan clone (t002, t010, t088, t149, t539 and t3824) and the *spa* type t037 was associated with BEC. These data was similar with studies that showed that in the last decade, the New York/Japan clone was highly prevalent in many Brazilian hospitals [20–23]. In contrast until 2005, MRSA isolates SCC*mec* Iva (57 %) and SCC*mec* type III belonged to and the *Brazilian Endemic Clonal Complex* (BECC) (39 %) were predominant in our hospital [23]. However, since 2010, there was a switched of pattern in our hospital, MRSA isolates SCC*mec* II (60 %) with characteristics of NY/Jpn clone were predominant instead of MRSA isolates belonged to BEC that represent only 10 % of isolates [22].

In our study, none of MRSA isolates associated with the NY/Jpn clone carry *tst* gene and 92 % harbored SCC*mec* II (only one isolate carried SCC*mec* I). Interestingly, all MRSA isolates with *tst* gene carried the same *spa* type and SCC*mec* (t0149 – SCC*mec* I) but belonged to the different PFT E and differ from Japanase studies [24, 25]. The major MRSA distributed among Japanese hospitals is New York / Japan clone with SCC*mec* II and generally carries the *tst* gene [24, 25]. However, American studies showed that isolates of MRSA USA100 (associated with NY/Jpn clone) harbor SCC*mec* II and rarely carry *tst* gene [24–26]. In view of these results, our MRSA isolates belonged to NY/Jpn clone seen be more similar to the USA100 MRSA isolates.

In the present study, only 4 (12,5 %) isolates showed a pattern closely related to the BEC. All of them had the same *spa* type (t037) and SCC*mec* type III. About the virulence profile, one isolate did not have the same pattern of virulence than otherisolates. Although there are few studies with *spa* typing in Brazil, our findings are similar to other Brazilian studies that also found that MRSA isolates t037-SCC*mec* III were strongly related with Brazilian epidemic clone [27, 28]. On the other hand, a new *spa* type t14789 was identified in our study in a cirrhotic patient as well as 6 *spa* types t010, t088, t149, t311, t539 e t3824 that were identified for the first time in Brazil.

This study has several limitations such as it was performed in only one center, the low number of MRSA infection in the post liver transplantation patients did not allow us to compare the virulence among colonization and infection strains and to conduct an intervention study with decolonization with mupirocin and chlorhexidine bath. Unfortunately, we could not perform MLST for all isolates. However, among the STs identified in our study, ST5, ST105, ST239 and ST1176 had already been reported in Brazil [21–23, 27–29], including studies in our Hospital [22, 23]. Nevertheless it was the first description of ST1510 in Brazil.

In this study, PFGE had a greater discriminatory power than *spa* typing. Furthermore, isolates within the same PFGE cluster had different *spa* types. Golding and colleagues [10] developed a large study in Canada with MRSA isolates and showed that *spa* types were not limited to a specific PFGE pattern, and that indistinguishable PFGE patterns could be of different *spa* types. These authors made a more detailed analysis of SSR based on the degree of similarity of *spa* types, and showed six-*spa* clonal complexes that were related to specific epidemic clones. Thus, it is possible to suggest that further studies on the similarity between the SSR are needed to establish a more reliable correlation between PFGE patterns [10].

## Conclusion

In conclusion, a high proportion of patients were colonized with MRSA and nares were the most frequent site of colonization. Liver-transplantation patients were more frequently colonized by MRSA than cirrhotic patients. SCC*mec* type II was the most frequent SCC*mec*, and the predominant clone was related to the NY/Jpn cluster. PFGE showed a greater discriminatory power than *spa* typing. We identified a new *spa* t14789 and *spa* types and ST never reported in Brazil.

## Methods

### Ethics statement

The study was performed from August 2010 to December 2011 in a university hospital located in São Paulo, Brazil, the Central Institute of Hospital das Clínicas of University of São Paulo (ICHC-FMUSP). All patients filled out a written form agreeing to participate in this study, which was approved by the ethics committee of “Hospital das Clinicas” and Medical University of Sao Paulo. The approval number is 0307/09.

### Study design

Prospective observational study, one isolate per patient was selected for further molecular typing. When patients showed MRSA isolates from the both sites with the same PFGE, the nasal MRSA isolate was choose for next steps.

### Population

Nasal and groin swabs were collected from patients on the waiting list for liver transplantation who came to medical appointments or were hospitalized in the liver transplant unit. Swabs of liver-transplanted patients were also collected on the first day after to surgery or within 48 h after transplantation. Data from nosocomial infections due to MRSA during hospitalization and until 30 days post-transplantation were evaluated.

All patients agreed to participate in the 335 study. Demographic and clinical data of patients were collected by a prior questionnaire and chart reviews. A data base was build using Epi Info (version 3.5.1), and proportion of MRSA colonization among cirrhotic and liver transplantation patients was compared using chi-square test, p value <0.05 was consider significant.

### MRSA identification

The swabs (BAC SWAB 1002 DME®) were cultured in accordance with protocols previously described [30, 31]. Strains were identified as MRSA according to screening Oxacillin resistance as recommended by CLSI (*Clinical Laboratory Standards Institute)* 2010.

### DNA extraction

The DNA genomic extraction was performed by Illustra Bacteria Genomicprep Mini Spin® (Ge Healthcare Life Science, USA) according to the manufacturer’s instructions.

### Multiplex PCR for Detection of *mecA* and *coA* genes

A multiplex PCR (M-PCR) was performed to confirm methicillin resistance of the *S. aureus* isolates. The M-PCR amplifies the coagulase gene *coA* (intrinsic to *S. aureus*) and the *mecA* gene which confers resistance to methicillin. PCR was performed according protocol described previously [32].

### Determination of *SCCmec*

The determination of *SCCmec* types was performed using the M-PCR method as described by Zhang et al. [6], with this method it was possible to determine SCC*mec* types I, II, III, IVa, IVb, IVc, IVd and V.

### Virulence factors

Specific genes (*clf*B, *fib*, *fnb*PA, *eta*, *etb*, *luk*-DE, *pvl* and *tst*) which encode adhesins and toxins in *S. aureus* were identified by M-PCR according to the protocol described previously [33].

### Spa typing

The amplification of the polymorphic region X of the *spa* gene was performed with chromosomal DNA purified from each isolate using primers designed by Shopsin and colleagues [8]. The amplified products were sent to the Human Genome Research Center of University of São Paulo for sequencing. The sequences were analyzed using Bioedit software (version 7.2.5; Ibis Biosciences, CA). Lastly, the *spa* type was determined using the online software spaTyper (http://spatyper.fortinbras.us/) and confirmed on SpaServer database (http://www.spaserver.ridom.de/) in the National Microbiology Laboratory of Canada, Winnipeg, Manitoba, Canada.

### PFGE

The chromosomal DNA of MRSA isolates was digested using the restriction enzyme *Sma*I (Amersham Pharmacia Biotech, USA). Pulsed-field gel electrophoresis was performed using a CHEF DR-II system (Bio-Rad, USA) according to previously described [34]. PFGE patterns were analyzed in Bionumerics version 7.1 (Applied-Maths, Sint-Martens-Latem, Belgium). DNA fragments were manually curated and normalized using the molecular weight standard run on each gel. A 1,5 % band tolerance and optimization of 0,5 % was selected for use during comparisons of DNA profiles as suggested by Mulvey and colleagues [35]. Cluster analysis was performed by the unweighted pair group method using arithmetic averages (UPGMA). Isolates were considered to be genetically related if Dice coefficient correlation was >80 %. DNA fragments of the strains BK2464 and HSJ216 (representative of New York/Japan epidemic clone and Brazilian epidemic clone, respectively) were included to comparisons of DNA profiles according to PFGE patterns previously generated by Oliveira and colleagues [34].

### MLST

Nine isolates were selected, one isolate of each PFT to performed MLST profiles according to procedures published elsewhere [36]. The sequences of seven housekeeping genes (*arc*C, *aro*E, *glp*F, *gmk*, *pta*, *tpi* and *yqi*L) were compared to existing sequences in the MLST database (http://www.mlst.net) for the assignment of allelic numbers. Sequence types (ST) were assigned according to their allelic profiles.

## Abbreviations

*S. aureus*: *Staphylococcus aureus*
MRSA: Methicillin-resistant *Staphylococcus aureus*
PVL: Panton-Valentine leucocidin
SCC*mec*: Staphylococcal Chromosome Cassette *mec*
PCR: Polymerase Chain Reaction
MLST: Multi Locus Sequence Typing
ST: Sequence type
PFGE: Pulsed-Field Gel Electrophoresis
PFT: Pulsed-Field type
SSR: Short-sequence repeat
NY/Jpn clone: New York/Japan epidemic clone
BEC: Brazilian epidemic clone
DNA: Deoxyribonucleic acid
M-PCR: Multiplex PCR
